# Role of deformation imaging in left ventricular non-compaction and hypertrophic cardiomyopathy: an Indian perspective

**DOI:** 10.1186/s43044-020-0041-z

**Published:** 2020-01-22

**Authors:** A. J. Ashwal, Sudhakar Rao Mugula, Jyothi Samanth, Ganesh Paramasivam, Krishnananda Nayak, R. Padmakumar

**Affiliations:** 1 Department of Cardiology, Kasturba Medical College Manipal, Manipal Academy of Higher Education(MAHE), Manipal, India; 2 Department of Cardiovascular technology, Manipal College of Health Professions, Manipal, Manipal Academy of Higher Education(MAHE), Manipal, India

**Keywords:** Speckle tracking, non-compaction, rotation mechanics, strain

## Abstract

**Background:**

Speckle tracking echocardiography (STE) has emerged as a novel feasible tool for the assessment of left ventricular rotational parameters. Since hypertrophic cardiomyopathy(HCM) shares morphologic features with left ventricular non-compaction (LVNC), we used this imaging modality to compare rotational mechanics between these two entities.

**Results:**

We compared global and regional LV function and rotational mechanics between LVNC, HCM, and healthy subjects using STE. Longitudinal strain and torsion were obtained from echocardiographic images from parasternal short axis as well as standard LV apical views. Twelve patients with LVNC [mean age 46.12 ± 14.66 years; median 47.5 IQR (39.25–58.5) years] were compared with 18 HCM patients [mean age 49.48± 17.22 years; median 56 IQR (33–65) years] and 18 healthy subjects [mean age: 51.50± 12.51 years; median 51(45.75-58) years]. LVNC group showed a significantly reduced longitudinal strain at the apical region compared to HCM group (− 12.18 ± 6.25 vs − 18.37 ± 3.67; *P* < 0.05). Rigid body rotation(RBR) was found in 50% of patients whereas the other half had a normal rotation at the apex and the base. Among the patients with RBR, all patients had a uniform counterclockwise rotation.

**Conclusion:**

Longitudinal strain was impaired in both the forms of cardiomyopathy; however, LVNC showed a more significant reduction in the apical region compared to patients with HCM suggesting a development abnormality in these regions. A reduction in left ventricular torsion was specifically noted among patients with LVNC with a uniform anticlockwise rotation of LV base and apex.

## Background

Left ventricular non-compaction (LVNC) is a rare cardiac anomaly presenting commonly as left ventricular systolic dysfunction. Though cardiac magnetic resonance imaging has become a standard investigation for the diagnosis, newer echocardiographic methods like strain, strain rate and torsional analysis play an important role in assessing regional cardiac function in left ventricular non-compaction and differentiating it from similar phenotypes like dilated cardiomyopathy and HCM. LVNC can occur in both sporadic and familial forms [1]. Familial forms share genetic mutations with HCM including mutations in genes encoding for sarcomeric proteins. Morphologically, HCM may resemble LVNC by the presence of trabeculations and crypts. Unlike LVNC where the crypts are typically located in the distal portion of LV chamber and not penetrating the wall of normal myocardium, crypts in HCM are situated in the basal part of LV chamber [2]. LVNC can also present with increased wall thickness similar to HCM; however, this phenotype is associated with poor prognosis. It is important to differentiate LVNC from HCM as the incidence of ventricular arrhythmias are higher in LVNC. We demonstrated the usefulness of speckled tracking echocardiography to assess the regional cardiac function and left ventricular twist and importance of these echocardiographic parameters in differentiating between LVNC and HCM.

## Methods

In this observational study, we collected stored echocardiographic data retrospectively during the echocardiographic evaluation done between January 2016 and March 2018 at a tertiary care hospital in South Karnataka, India. We collected available data from 12 consecutive patients with left ventricular non-compaction, eighteen cases of hypertrophic cardiomyopathy and eighteen healthy controls. Inclusion criteria included patients in sinus rhythm, echocardiographic criteria that met a diagnosis of LVNC and adequate echocardiographic image quality that allowed for a complete assessment of LV myocardial mechanics. Known cases of ischemic heart disease, primary valvular heart disease, previous cardiac surgery, and poor echocardiographic images were excluded from the study. Controls included individuals who were healthy, normotensive, not on any medications and who had complete normal echocardiograms. The study was approved by the institutional ethics committee.

The diagnosis of isolated LVNC was based on the presence of the following criteria: (1) visual appearance of two distinct compacted epicardial layer and a non-compacted endocardial layer; (2) marked trabeculation and deep intertrabecular recesses within the non-compacted layer; (3) non-compacted to compacted end-diastolic myocardial ratio of > 2 and (4) no evidence of congenital or acquired heart disease [3]. Cardiac magnetic resonance imaging was done in five patients with LVNC where there was a disparity in echocardiographic diagnosis.

The diagnosis of HCM was fulfilled by an otherwise unexplained hypertrophied LV with a maximal ventricular septal wall thickness ≥ 15 mm and a septal to posterior wall ratio of more than or equal to 1.4 [4]. Based on the echocardiographic examinations, HCM was classified into four categories as follows:

- Type 1: hypertrophy involving interventricular septum (IVS) and anterior wall (AW) of LV.
- Type 2: hypertrophy involving IVS, AW, and lateral wall (LW) of LV.
- Type 3: hypertrophy involving apex only (excluded in the current study as would not be reliable to compare LVNC and HCM involving the apex only)
- Type 4: concentric HCM, involving all the segments of LV.

### Echocardiographic data

Stored echocardiography images from Vivid 7 GE Healthcare system were analyzed offline using Echo Pac software (GE healthcare). Left ventricular ejection fraction (EF) was calculated by modified Simpson’s biplane method. Diastolic function was evaluated by e’ (early diastolic annular velocity) obtained from tissue Doppler imaging in which sample volume was placed at septal and lateral LV annulus [5]. Average left ventricular S (systolic annular velocity) was calculated for assessing left ventricular systolic function. Echocardiographic images from the parasternal short axis views at the basal, mid, and apical levels and from the 3 standard LV apical views (4-, 2-, and 3-chambers) were used to generate speckle tracking derived longitudinal strain and twist with breath-hold and stable electrocardiographic recording. Longitudinal strain demonstrates the percentage change in the myocardial length during the cardiac cycle, whereas the strain rate is the ratio of myocardial velocity gradient to the distance between two myocardial segments. Left ventricular twist refers to the wringing motion of the heart along its long axis with relative rotation between basal and apical LV segments. Myocardial trabeculations were defined as localized protrusions of the endocardial surface ≥ 3 mm in diameter, associated with intra trabecular recesses on 2-D echocardiography. Myocardial longitudinal strain and strain rate were obtained by speckle tracking technique from the 3 apical views at frame rate > 50/s. The region of interest was traced in the compact part of the myocardium [6]. LV global longitudinal strain was averaged from peak longitudinal strains in a 16 segments LV model [7]. LV longitudinal basal and mid-LV strain (6 segments each) and longitudinal apical strain (4 segments) were averaged and analyzed separately. LV rotation was assessed in the end-diastolic frame in each parasternal short-axis image. Tracking points were separated about 60° from one another so as to fit total LV circumference and then according to American Society of Echocardiography (ASE) consensus, a positive value was assigned to counterclockwise rotation and a negative value to clockwise rotation. First, the peak systolic rotation was measured at the apex following which basal rotation was measured at a time interval synchronous with peak systolic apical rotation. Torsion was measured as peak apical rotation minus basal rotation. We also analyzed the direction of rotation both in isovolumic contraction and rest of the systole and identified rotation either as normal or rigid body rotation, which was entirely clockwise or counterclockwise at both the base and the apex during ejection phase of systole. Offline analysis was analyzed by a single experienced echocardiographer who was not involved in image acquisition and had no knowledge of other echocardiographic variables.

### Statistical analysis

Parametric data were presented as mean ± standard deviation. One-way analysis of variance (ANOVA) was used to find the mean difference between three independent groups with Bonferroni normalization for *P* value. Kruskal-Wallis test was used when variables were not normally distributed. Statistical significance was defined as two-tailed *P* value < 0.05.

## Results

### Baseline transthoracic echocardiographic findings

The mean age was 46.12 ± 14.66[median 47.5 IQR (39.25–58.5)] years in patients with LVNC whereas 49.48 ± 17.22[median 56 IQR (33–65)] years and 51.50± 12.51[median 51(45.75–58)] years in patients with HCM and controls respectively (Table 1). Among the patients with HCM, concentric hypertrophy was observed only in one patient whereas type 2 was observed in nine patients. There were four patients identified in whom systolic anterior motion of mitral valve (SAM), left ventricular outflow tract obstruction (LVOT), and mitral regurgitation (MR) were noted during echocardiography. The mean left ventricular ejection fraction (EF) was 30.38 ± 12.24% in LVNC and 71.33 ± 5.53% in the HCM group (Table 1). LV global systolic functions by EF and by average LV S (mitral tissue annular velocity) were reduced in LVNC compared to healthy individuals and HCM patients (*p* < 0.05); however, systolic function did not differ between healthy controls and HCM patients (Table 1). Diastolic function as assessed by average Em (early diastolic annular velocity) and Am (late diastolic annular velocity) both of which were significantly lower in LVNC and HCM compared with controls (Table 1).

**Table 1:** Baseline demographic and echocardiographic findings in patients with LVNC, HCM, and controls

Parameter	Control		LVNC		HCM		*P* value ANOVA
	Mean	SD	Mean	SD	Mean	SD	
Age	51.50	12.51	46.12	14.66	49.48	17.22	0.705
ALEF	67.22	3.68	30.38	12.24*	71.33	5.53^$^	<0.001
IVS_Em	0.13	0.16	0.08	0.02	0.09	0.14	0.664
IVS_Am	0.09	0.01	0.04	0.01*	0.07	0.02*^$^	<0.001
LW_Em	0.11	0.03	0.09	0.03	0.08	0.02*	0.06
LW_Am	0.09	0.02	0.04	0.01*	0.07	0.03*	<0.001
Em_avg	0.10	0.02	0.07	0.01*	0.07	0.02*	0.006
Am_avg	0.10	0.02	0.04	0.01*	0.08	0.02*^$^	<0.001
S_LV_avg	0.08	0.01	0.05	0.01*	0.07	0.02^$^	0.001

*LVNC* left ventricular non compaction, *HCM* hypertrophic cardiomyopathy, *SD* standard deviation, *ANOVA* analysis of variance, *ALEF* area length ejection fraction, *IVS_Em* early diastolic mitral velocity at septal annulus level, *IVS_Am* late diastolic mitral velocity at septal annulus level, *LW_Em* early diastolic mitral a velocity at lateral annulus wall level, *LW_Am* late diastolic mitral velocity at lateral annulus level, *Em_avg* average early mitral velocity, *Am_avg* average late mitral velocity, *S_LV_avg* global average left ventricular strain

*P < 0.05 vs controls

^$^*P* < 0.05 vs LVNC

### Global LV function: deformation imaging

Tissue deformation imaging showed significant decrease in global strain (9.20 ± 4.37 vs 18.83 ± 2.38; *P* < 0.05) and systolic strain rate (0.68 ± 0.15 vs 0.88 ± 0.16; *P* < 0.05) among both LVNC and HCM groups. Early and late diastolic strain rates were also substantially reduced among these groups (Table 2).

**Table 2:** Longitudinal deformation parameters in LVNC, HCM, and controls

Longitudinal deformation parameters	Control	LVNC	HCM	*P* value ANOVA
Global strain (%)	23.24 ± 1.45	9.20 ± 4.37^*^	18.83 ± 2.38^*$^	<0.001
Global SSR (/s)	1.30 ± 0.10	0.68 ± 0.15^*^	0.88 ± 0.16^*$^	<0.001
Global ESR (/s)	1.36 ± 0.18	0.83 ± 0.24^*^	0.88 ± 0.17^*^	<0.001
Global LSR (/s)	1.36 ± 0.12	0.83 ± 0.32^*^	0.85 ± 0.15^*^	<0.001

*LVNC* left ventricular non compaction, *HCM* hypertrophic cardiomyopathy, *SD* standard deviation, *ANOVA* analysis of variance, *SSR* systolic strain rate, *ESR* early diastolic strain rate, *LSR* late diastolic strain rate

*P < 0.05 vs controls

^$^*P* < 0.05 vs LVNC

#### Regional systolic and diastolic function

Longitudinal strain at both basal (− 7.74 ± 3.65 vs − 18.57 ± 3.04) and apical level (− 12.18 ± 6.25 vs − 18.37 ± 3.67) was reduced in LVNC and HCM compared to controls (*P* < 0.05) (Table 3). Similarly, systolic strain rate (SSR) was significantly lower in LVNC and HCM compared to healthy controls (Table 2). When longitudinal strain was compared between apical and basal segment, it was observed that the LVNC group manifested significant decrease in longitudinal strain from apex to base as compared with HCM and control group which did not show any change (Table 3).

**Table 3:** Speckled tracking echocardiographic parameters in LVNC, HCM, and controls

Parameter	Control		LVNC		HCM		*P* value ANOVA
	Mean	SD	Mean	SD	Mean	SD	
BASAL_LONG_STRAIN	− 23.83	2.71	− 7.74	3.65*	− 18.57	3.04*^$^	<0.001
MID_LONG_STRAIN	− 22.89	1.69	− 7.68	3.90*	− 19.54	3.08*^$^	<0.001
APICAL_LONG_STRAIN	− 23.00	2.08	− 12.18	6.25*	− 18.37	3.67*^$^	<0.001
BASAL_LONG_SSR	− 1.30	0.19	− 0.70	0.27*	− 0.95	0.24*^$^	<0.001
MID_LONG_SSR	− 1.33	0.21	− 0.54	0.13*	− 0.82	0.25*^$^	<0.001
APICAL_LONG_SSR	− 1.27	0.21	− 0.79	0.26*	− 0.89	0.24*	<0.001
BASAL_LONG_ESR	1.37	0.28	0.74	0.25*	0.72	0.30*	<0.001
MID_LONG_ESR	1.37	0.22	0.69	0.21*	0.98	0.22*^$^	<0.001
APICAL_LONG_ESR	1.34	0.27	1.10	0.43	0.67	0.19*^$^	<0.001
BASAL_LONG_LSR	1.45	0.30	0.67	0.14*	0.94	0.30*	<0.001
MID_LONG_LSR	1.34	0.14	0.58	0.14*	0.63	0.17*	<0.001
APICAL_LONG_LSR	1.28	0.20	1.24	1.03	0.97	0.32	0.156

*LVNC* left ventricular non compaction, *HCM* hypertrophic cardiomyopathy, *SD* standard deviation, *ANOVA* analysis of variance, *LONG* longitudinal, *SSR* systolic strain rate, *ESR* early diastolic strain rate, *LSR* late diastolic strain rate

*P < 0.05 vs controls

^$^*P* < 0.05 vs LVNC

Early diastolic strain rate (ESR) was impaired both in LVNC and HCM group compared with healthy controls suggesting impaired diastolic relaxation in these two groups compared with controls (Table 2).

#### Torsion analysis

**Fig. 1:**
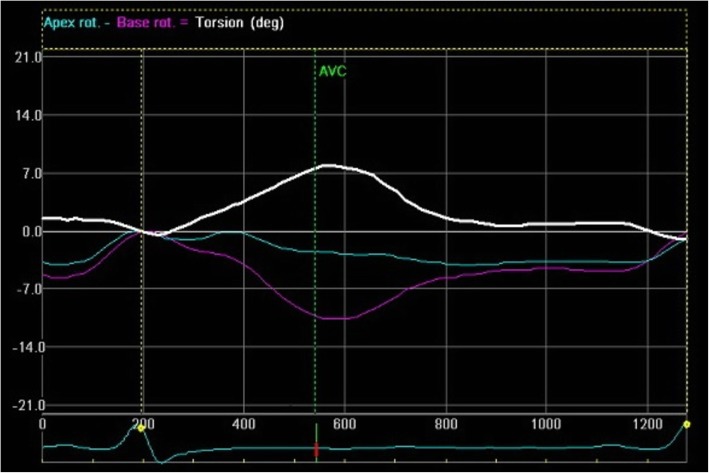
Speckle tracking echocardiography (Echo Pac software) in patients with LVNC showing net basal rotation (purple color) and net apical rotation (green color) in the same counter-clockwise direction. Net torsion is displayed in white color

**Fig. 2:**
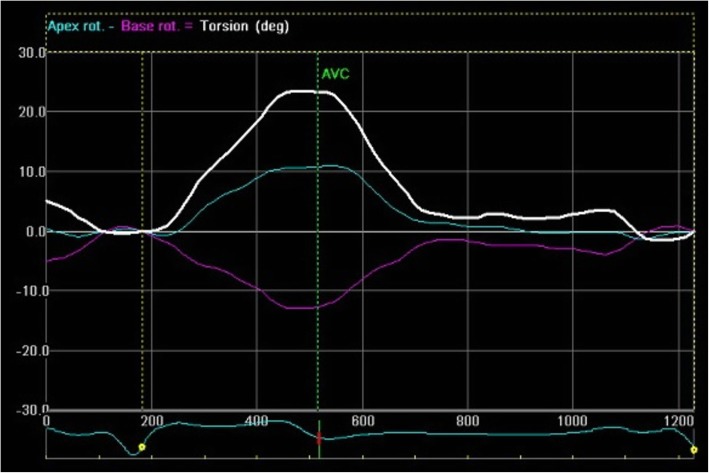
Speckle tracking echocardiography (Echo Pac software) in patients with HCM showing net basal rotation (purple color) in counter-clockwise direction and net apical rotation (green color) in clockwise direction. Net torsion is displayed in white color

**Fig. 3:**
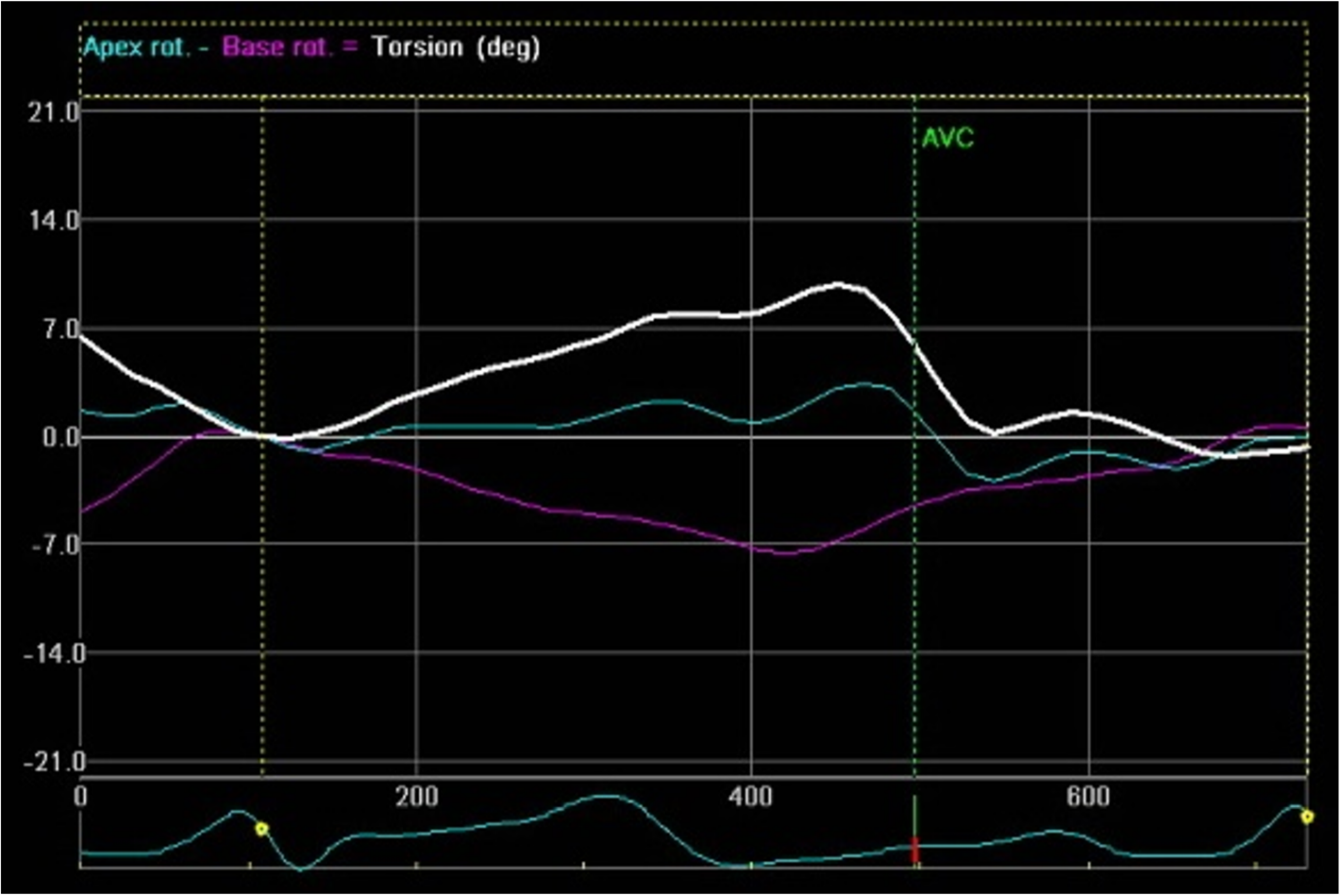
Speckle tracking echocardiography (Echo Pac software) in normal subjects showing net basal rotation (purple color) in counter-clockwise direction and net apical rotation (green color) in clockwise direction. Net torsion is displayed in white color

A normal pattern of LV rotation was found in six (50 %) patients with LVNC whereas rigid body rotation was noted in remaining patients (Fig. 1). A uniform counter clockwise rotation was found in all patients with rigid body rotation. All except one patient with hypertrophic cardiomyopathy had normal basal counterclockwise and apical clockwise rotation (Fig. 2). The remaining one patient had both basal and apical clockwise rotation. All controls had a normal pattern of rotation at the apex and the base (Fig. 3). A reduction in apical rotation and torsion was specifically observed in the LVNC patients compared with controls (Table 4). Also patients with LVNC had a significantly reduced apical rotation compared with HCM. Patients with HCM had significantly higher torsion compared with healthy controls. However, there was no difference noted in basal rotation among the three groups (Table 4).

**Table 4:** Myocardial deformation parameters in LVNC, HCM, and controls

Parameter	Control median (IQR)		LVNC median (IQR)		HCM median (IQR)		*P* value ANOVA
	Mean	SD	Mean	SD	Mean	SD	
BASAL_ROTATION	− 3.82	2.40	− 4.40	2.71	− 5.05	3.90	0.496
	− 3.44 (− 4.85, − 1.99)		− 4.64 (− 4.64, − 2.83)		− 4.64 (− 8.16, − 3.18)		
APICAL_ROTATION	6.97	3.33	− 0.17	5.34*	10.84	6.89^$^	<0.001
	6.19 (3.86, 9.49)		− 0.43 (− 5.38, + 5.46)		8.59 (4.64, 16.36)		
TORSION	10.41	4.72	3.69	5.14*	15.86	7.28*^$^	<0.001
	8.85 (6.55, 13.49)		4.81 ( − 1.24, + 8.12)		14.61 (8.51, 23.09)		

*LVNC* left ventricular non compaction, *HCM* hypertrophic cardiomyopathy, *SD* standard deviation, *ANOVA* analysis of variance, *IQR* inter quartile range

*P < 0.05 vs controls

^$^*P* < 0.05 vs LVNC

## Discussion

Left ventricular non-compaction is a rare congenital heart disease which develops as a consequence of absent or incomplete myocardial compaction during embryonic morphogenesis. Though two-dimensional echocardiography is the standard diagnostic tool for LVNC, cardiac MRI is useful to confirm or rule out LVNC when the apex is difficult to visualize. Normally, compaction progresses from epicardium to the endocardium and from the base towards the apex of the heart. LVNC is often accompanied by early development of systolic or diastolic dysfunction which may lead to heart failure and ventricular arrhythmias. Hypertrabecularization of the myocardium significantly increases the risk of systemic embolization as well [8].

Our present study showed decrease in global LV function as suggested by global strain and strain rate analysis among both LVNC and HCM subjects, being worst among non-compaction cases. However, diastolic function was reduced in both in comparison to controls with no significant intergroup difference. Regional deformation analysis showed presence of apico-basal gradient in longitudinal strain among LVNC, which was not evident among HCM subgroups. Various studies focusing on these populations have shown wide range of results [6, 9, 10]. In contrast to our findings, study by Haland et al. showed that LVNC exhibits homogenous reduction in longitudinal strain across basal and apical LV regions with no apico-basal gradient. This pattern of reduced apical function compared to basal segments in patients with non-compaction is possibly attributed to embryogenic theory which states that compaction of myocardium occurs from epicardium to endocardium and from base to the apex [1]. Though HCM manifests decreased global longitudinal strain, apico-basal LV segmental gradient was preserved in our study. This is in contrast to the study by Haland et al. which showed preserved apical and more reduced basal function in HCM.

In the normal heart, the LV base rotates clockwise while the apex rotates counter-clockwise during the systole, producing a “towel-wringing” motion of the heart. The net difference between LV base and the LV apex is called “net twist angle.” This twist is due to dynamic interaction of subepicardial and sub endocardial myocyte helices. Peak LV systolic twist is directed by sub-epicardial fibers due to their longer arm of movement whereas early LV systolic twist in opposite direction is directed by sub endocardial helix of myocardial fibers during isovolumic contraction. The LV twist represents a phenomenon that links systolic contraction with diastolic relaxation [11].

Speckle-tracking echocardiography can be used to accurately assess LV twist. Van Dalen et al. were the first using 2DSTE to demonstrate that the direction of LV basal rotation and that of LV apical rotation are the same, resulting in near absence of LV twist in patients with LVNC known as “LV solid/rigid body rotation” [12]. Varying degrees of maturation of two helices at the apical and basal region could be postulated for rigid body rotation.

To our knowledge, this is the only study conducted so far comparing left ventricular rotational mechanics in patients with left ventricular non-compaction, hypertrophic cardiomyopathy, and healthy subjects. A normal pattern of LV rotation was found in half of our patients whereas rigid body rotation was found in rest half of patients. F Peters et al. and Van Dalen et al. showed prevalence of rigid body rotation as 53.3 % and 83 %, respectively [12, 13]. The following reasons can be implicated in the lower prevalence of RBR in our study compared to Van Dalen et al. LVNC may be caused by different genetic profiles which may be affected by race and population studied and hence the manner of remodeling may be different. Selection bias may also have contributed to the differences involved. Van Dalen et al. included 17 patients with familial LVNC in their study of all whom showed RBR. However, in our study, patients were mostly non-familial LVNC (demonstrated by negative family history and absence of non-compaction on echocardiography in first degree relatives). Finally, referral bias would have contributed to the results. As most of the patients included in our study were symptomatic, our patients had a much lower ejection fraction compared to Van Dalen’s cohort. Uniform clockwise rotation of LV segments had assumed to be a hallmark feature of LVNC in most of the studies. In this study, unidirectional rotation in anticlockwise direction was found particularly among LVNC subjects, whereas two distinct direction of rotation at base and apex were noted among HCM and control groups. The exact mechanism of this counter-clockwise rotation of LV segments in our study is still unknown.

In this study, apical rotation and torsion was significantly reduced specifically among LVNC subjects in comparison to other two groups, whereas HCM showed increased torsion values in comparison to controls. F Peters et al. showed a significant lower apical and basal rotation in LVNC compared to controls. A decrement in both apical and basal rotation may be due to either a decrease in function of the subepicardial fibers or a combination of both helices having decreased function. Our study demonstrated that torsion in patients with HCM was higher compared to healthy subjects. Similar results were found in the study conducted by Young et al. and Saito et al. [14, 15]. Prinz et al. studies pediatric patients diagnosed with HCM and showed significant increase in torsion compared to healthy children [16]. In healthy heart, counter clockwise rotation of the subepicardial fibers dominates clockwise rotation of the sub endocardial fibers (due to larger radius). An increased torsion in patients with HCM is the result of increased lever arm (to subepicardial fibers) due to concentric hypertrophy.

Few limitations of this study warrants consideration. First, as it was a retrospective echocardiographic study, clinical variables like NYHA class, systolic and diastolic blood pressure, heart rate, and usage of cardiac drugs were not taken into account. Secondly, because of the retrospective nature of the study, the influence of various loading conditions like preload, afterload and contractility on LV twist was not documented. The presence and severity of LV dyssynchrony which can contribute to remodeling and ejection fraction was not taken into account. However, stable electrocardiographic recording showed that none of these patients had QRS duration more than 130 ms. We did not study the long-term follow-up of these patients which can provide information on the clinical consequences. Long-term follow-up is needed to know if patients with reduced torsion progress to RBR or if patients with RBR have unfavorable clinical consequences compared to patients with only reduced torsion.

## Conclusion

Longitudinal strain was impaired in both the forms of cardiomyopathy; however, LVNC showed a more significant reduction in the apical region compared to patients with HCM suggesting a development abnormality in these regions. A reduction in left ventricular torsion was specifically noted among patients with LVNC with a uniform anticlockwise rotation of LV base and apex. Patients with HCM had a higher torsion compared to healthy subjects. The study coveys that the use of speckle tracking echocardiography has a role in differentiating HCM from LVNC.

## Data Availability

The datasets used and/or analyzed during the current study available from the corresponding author on reasonable request.

## Abbreviations

HCM: Hypertrophic cardiomyopathy
IQR: Interquartile range
LVNC: Left ventricular non-compaction
RBR: Rigid body rotation
STE: Speckle tracking echocardiography
